# “They should be like penicillin”: barriers to the integration of medications for opioid use disorder in specialty treatment programs

**DOI:** 10.1186/s13722-025-00633-3

**Published:** 2025-12-05

**Authors:** Isha K. Desai, Kathryn Burke, Jewyl Raikes, Justin Xu, Yuzhong Li, Brendan Saloner, Kenneth A. Feder, Noa Krawczyk

**Affiliations:** 1 Department of Health Policy and Management, Johns Hopkins Bloomberg School of Public Health, 615 N Wolfe Street, Baltimore, MD 21205 USA; 2 Department of Health Policy and Management, George Washington University, 950 New Hampshire Ave, #2, Washington, DC 20052 USA; 3 New York University School of Global Public Health, 708 Broadway, New York, NY 10003 USA; 4 Department of Mental Health, Johns Hopkins Bloomberg School of Public Health, 615 N Wolfe St, Baltimore, MD 21205 USA; 5 Center for Opioid Epidemiology and Policy, NYU Grossman School of Medicine, 550 1st Ave, New York, NY 10016 USA

**Keywords:** Opioid use disorder, Medication treatment, Barriers to treatment, Buprenorphine, Specialty treatment facility

## Abstract

**Background:**

Specialty drug treatment programs should be a key setting to treat opioid use disorder (OUD), but most programs continue to not treat OUD patients with evidence-based medications for opioid use disorder (MOUD). COVID-19 introduced some flexibilities that could improve uptake of MOUD but ongoing barriers have not been examined. We examined barriers and facilitators to integrating MOUD post-COVID among specialty treatment programs in New Jersey.

**Methods:**

Between March-July 2023, we conducted 14 semi-structured qualitative interviews with leadership and clinical staff of New Jersey specialty outpatient drug treatment programs, with varying levels of client MOUD uptake. Thematic analysis examined barriers and facilitators to providing MOUD in specialty treatment programs in the post-COVID-19 era.

**Results:**

Treatment providers revealed that financial barriers, including gaps in insurance coverage after COVID Medicaid expansions were terminated, continue to prevent MOUD use. Second, despite new buprenorphine telehealth flexibilities, a shortage of buprenorphine providers continues to limit ability to offer MOUD. Third, stigma against MOUD among clients, families, providers, and ancillary service providers such as housing programs continues to prevent MOUD initiation. Successful facilitators include expanding educational efforts on effectiveness of MOUD and harm reduction for clients and community members, forming specialized partnerships with pharmacies, and expanding care coordination efforts for those on MOUD.

**Conclusions:**

Despite efforts by federal and state governments, MOUD continues to be underutilized in specialty treatment settings, introducing significant risks for patients. Efforts should focus on reducing gaps in insurance coverage, improving provider knowledge of regulatory changes around MOUD, and supporting anti-stigma campaigns.

## Background

Treatments for opioid use disorder (OUD) that include medications (MOUD) – methadone, buprenorphine, and extended-release naltrexone – are associated with reduced overdose risk [1, 2], acute care outcomes [2], illicit opioid use [3, 4], infectious disease transmission and criminal activity, and increased retention in treatment [1, 4]. In New Jersey, the fatal overdose rate for all opioids increased during the Covid-19 pandemic (27.5 per 100,000 in 2020 to 28.5 per 100,000 in 2022), but decreased to 24.8 per 100,000 in 2023 [5]. As individuals receiving MOUD treatment are at lower risk of overdose, supporting the increased uptake of these medications in treatment plans remains vital.

Specialty substance use disorder (SUD) treatment programs, which encompass outpatient and residential programs subject to state licensure and regulation, have historically been the primary providers of SUD treatment services in the U.S. Adoption of MOUD in specialty SUD treatment settings has been a longstanding challenge. As recently as 2022, fewer than 60% of specialty substance use treatment programs even offered MOUD [6]. Even when these programs do offer them, most clients with OUD still do not receive medications [6, 7]. 

Some of this shortfall results from rules governing MOUD in the U.S.: methadone for OUD must be dispensed through federally certified and accredited Opioid Treatment Programs (OTPs). Further, until 2023, only physicians with a special waiver were able to prescribe buprenorphine, limiting the pool of trained buprenorphine providers. Finally, extended-release naltrexone (Vivitrol) is an opioid antagonist that may be administered by any qualified practitioner but is infrequently used because it requires patients to be fully abstinent from opioids to begin and continue treatment [8]. Past research has also pointed to inadequate insurance coverage; time-intensive program participation requirements; and stigma as contributing factors to low client uptake of MOUD [9, 10]. However, most past research has not focused specifically on the role of specialty substance use treatment programs.

In this study, we conduct an in-depth exploration of barriers and facilitators to providing MOUD among specialty SUD treatment programs in New Jersey. Leaders of programs that participated in a companion study of current practices around MOUD [11] were invited to complete interviews about perceived barriers to providing MOUD, and strategies that they believe have been effective at boosting MOUD uptake. Our goal was to inform policy makers and program leadership seeking to increase the use of MOUD in these programs.

## Methods

### Setting and recruitment

Our study was conducted in collaboration with the New Jersey’s Division of Mental Health and Addiction Services (DMHAS) as part of an ongoing effort to address overdose in the state. Participants were recruited from a previous sample of respondents for a companion survey in New Jersey that agreed to participate in a follow-up interview [11]. We used maximum variation sampling [12] to recruit 14 programs from four different categories of MOUD provision based on their survey responses: (1) “Have MOUD provider onsite and report more than half of clients receiving MOUD” (*n* = 3); (2) “Have MOUD provider onsite and report fewer than half of clients receiving MOUD” (*n* = 5); (3) “Have MOUD partner provider offsite” (*n* = 2); and (4) “Have no MOUD provider” (*n* = 4). Detailed responding program characteristics are shown in Table 1. Two research team members (ID and KB) initiated contact via email to recruit eligible respondents until the team agreed data saturation was reached. All study procedures were approved by the Johns Hopkins Bloomberg School of Public Health Institutional Review Board.

**Table 1:** Self-reported characteristics of participating specialty substance use treatment programs that are not opioid treatment programs (OTPs) (*n* = 14)

Characteristic	*N*	%
**Organization type**		
Non-profit	11	78.6
For-profit	3	21.4
**Provider availability**		
No provider onsite	4	28.6
Partner provider offsite	2	14.3
Provider onsite (low volume of MOUD clients)	5	35.7
Provider onsite (high volume of MOUD clients)	3	21.4
Type of MOUD provided*		
None provided	4	28.6
Methadone	2	14.3
Buprenorphine (Suboxone and Sublocade	10	71.4
Naltrexone (Vivitrol)	10	71.4
**Level of care**		
Standard or traditional non-intensive outpatient treatment	10	71.4
Long-term residential treatment	2	14.3
Short-term residential treatment	2	14.3
**Percent of clients accessing MOUD onsite**		
0%	6	42.9
1 to 25%	2	14.3
25% to 50%	3	21.4
51% to 75%	2	14.3
76% to 100%	1	7.1
**Percent of clients accessing MOUD offsite**		
0%	4	28.6
1% to 25%	7	50.0
25% to 50%	2	14.3
51% to 75%	1	7.1
76% to 100%	0	0.0

*Not mutually exclusive

### Interviews

We used the Consolidated Framework for Implementation Research (CFIR) to develop a structured interview guide [13]. The interview guide was developed to elicit themes adapted from CFIR domains with barriers and facilitators to MOUD provision as the primary intervention of interest. Since the primary aim of this study was to investigate the factors influencing incorporation of all types of available MOUD in treatment plans, we asked respondents about MOUD broadly, which we defined to them as including methadone, buprenorphine, and naltrexone. The interview guide covered topics such as attitudes toward MOUD, perceived barriers to providing MOUD for clients, and strategies to provide and connect clients to MOUD. Participant interviews lasted between 30 and 60 min and were conducted via Zoom between March 2023 and July 2023. Informed consent was obtained from respondents via Zoom before the interview began.

### Coding and qualitative analysis

All interviews were audio recorded, professionally transcribed, and reviewed for accuracy by the research team. The codebook was deductively constructed based on the CFIR interview guide, and additional inductive codes were added based on the themes that emerged from the interviews. The study team coded the interviews using Dedoose. Five of the 14 interviews (36%) were coded by all study team members to enhance intercoder reliability and the full team resolved any discrepancies and adapted the codebook per the findings from the pilot coding. The lead author (ID) conducted thematic analysis [14] of primary barriers and facilitators. Given the stratified sampling approach, variation in themes was assessed across program/provider type and level of MOUD provision. Table 2 provides detailed quotes for each theme identified in the analysis.

**Table:** Themes and quotes from semi-structured interviews with specialty treatment programs on barriers and facilitators to MOUD

*Themes*	*Quotes*
**Barriers**	
*Financial Barriers*	“The cost is really, really, extensive for people. They, they just can’t afford it. They can’t afford to keep up with it if they are taking it. And it’s just, its also a lot of red tape to get it because you have to find a prescriber who’s going to prescribe it and then make sure that they have it on site. And usually that means your insurance has to fill it and approve it, and then it somehow has to get to that prescriber to be injected.” *-Program with no MOUD provider* “Well, for, for my clients specifically, it’s getting it right. Cause they can’t, they’re not getting it here. Um, and that, and that’s not really, I wish they could get it here, right? It’s not, I don’t want you to think that, um, I’m opposed to it and therefore don’t, uh, you know, I just, I don’t, I can’t get a prescriber here, right? Um, the, there’s another program which I won’t name, uh, up the road that, you know, their budget’s probably 30 million. Uh, they can hire a full-time doctor, right? Uh, our budget’s not close to that. I can’t hire a full-time doctor. So I need someone who’s willing to come in for three, four hours a week to see patients prescribe Suboxone, if that’s appropriate. Um, and that prescriber’s very hard to find.” *-Program with no MOUD provider* “You release people from prison and jail with no insurance, like how can that be a thing? Even when they’re on heavy psych meds sometimes…Like you’re releasing these people, they can’t get their meds, they’re gonna be back and they’re gonna hurt someone in the meantime, you know? So that part seems crazy to me. Like why not get them started on Medicaid?” *-Program with MOUD provider onsite*,* fewer than half of clients receiving MOUD* “If they do have insurance, it’s, um, I mean either or it’s, it could be up to $1,400 a month. Uh, so someone who, you know, doesn’t have insurance at all, there’s just no hope. Um, cause they’re not gonna pay $1,400 a month. I wouldn’t either. I wouldn’t be able to afford that. Even with insurance, there are copays, so, um, like any other medication. So, a lot of clients simply can’t afford it…The clients that have, um, private insurance that may have higher copays are the ones that tend to have more of the resistance. Um, so clients with Medicaid who actually can have a lot of these things covered, um, will, you know, I mean, they can, they tend to be more agreeable, but, um, if they’re, if they’re stuck on not wanting to commit to what’s required, they’re not gonna take it either. But for the, um, for the privately insured clients, they tend to start complaining about, well, I’ve been here for a while and my individual session copay is $40 and I’m not paying $40. So it’s a real issue. And you know, we, we by law can’t excuse copays, so we try to work with them on payment plans, but, um, it’s, you know, and then also medication can have a huge copay, you know. So it’s really the, the insured, the private insured client that tends to have more of the issue.” *-Program with no MOUD provider* “If their maintenance of MAT is that they see the doctor, like they do phone conferences with the doctor and they come once a month for urine screens, I think their biggest barrier would be getting cut off on Medicaid. Now with the COVID restrictions being lightened, um, once upon a time throughout COVID, Medicaid did not cut anybody off because that was their rule. But now that they don’t have that, we see people getting cut off off Medicaid midweek, mid-month.” -*Program with MOUD provider onsite*,* fewer than half of clients receiving MOUD* “The biggest barrier that I’ve seen is insurance coverage. If they lose coverage or, you know, they deny certain medications like some, I’ve seen some cases where Vivitrol is denied because it’s more expensive, but they will allow the oral medication than naltrexone. So that’s typically the biggest barrier.” *- Program with MOUD partner provider offsite* “If we get a call from a patient that has no insurance and they meet certain criteria, I, I forget, I don’t know what the limits are, but you can’t make a certain amount of money in a month, you know what I mean? It has to be under a certain amount. Um, then the admissions people will do an application for presumptive eligibility, and then within about 48 hours they’ll have active Medicaid that’s retroactive to that first date. Um, so if they’ve already gotten a [presumptive eligibility] done and then I guess they get a letter like three months later saying, oh, we need this, I don’t know, some documentation from them and they don’t send it back. Then they terminate their insurance, but yet now they have to wait a whole another year to get another PE done. It’s like an emergency PE like what was that thing we used to have at the hospital system where you came in with no insurance, and they would put you on community care or something.” -*Program with MOUD provider onsite*,* fewer than half of clients receiving MOUD* “So recently, like when Covid came through, they lifted all Medicaid, lifted a lot of stuff where you could get several PEs in a year of presumptive eligibility. And now they just changed it back where they can only get one a year. And so these people are beat. What can you do? You know, so we try to help them through that. Like I’ll make calls to different Rite Aids and get good RX coupons, but it’s a problem, you know, if they can’t get more than one PE in a year and they’ve already gotten that, now they can’t get their meds, you know?” *- Program with MOUD provider onsite*,* more than half of clients receiving MOUD*
*Shortage of Buprenorphine Providers*	“No one is gonna get high off Suboxone. No one is gonna get high off Vivitrol. No one is gonna die from any of this stuff. Let them prescribe it. What’s the worst that happens? It’s diverted to another drug addict to help them get clean?” *- Program with MOUD partner provider offsite* “Yeah, and I’m really concerned about the DEA telehealth Suboxone and stimulant schedule; they’re gonna be limiting that by saying they have to be in person. I’ve had calls from people who their telehealth prescriber has just stopped seeing them because they don’t even wanna wait for the May 11th. But, you know, I have a client who is gonna drive three hours so that she can see our prescriber in person, cause she’s three hours away in northern New Jersey. So she’s gonna drive from Northern New Jersey to Cape May. Um, and I just think that’s sad. I mean, she’s been a client with us for years. She, you know, we monitor her urine. I mean, what’s there really to me is no point. I mean, she’s been compliant. We, we, she’s, we see her in person as clinicians, but that’s not gonna matter. Cause the doctor has to see her in person. So, yeah. I just think that’s awful for people have to worry about.” *-Program with no MOUD provider* “…if I can go to my doctor and get prescribed an opiate, I should be able to go to the doctor and get prescribed MAT and it should be just as easy. There should not be any additional training, should not be any additional licensing, not be any additional credentials.there is no reason for [MOUD] to be treated the way they are. They should be like penicillin or like insulin or like any other medication.” *- Program with MOUD partner provider offsite* “Even, you know, a client on methadone, I can’t accept a client on methadone in my program because of, you know, different DEA regulations and we’re not licensed with the DEA to be able to accept that. Right? So there are still barriers, uh, in the state, in the country, depending on where a client is and where they’re going that precludes them from, from accessing some care.” *-Program with no MOUD provider* “…just more funds [are needed] to be able to invest in not only the number of prescribers but the quality of prescribers, to retain them, to get them in the door but then to retain them. Because it’s also hard when we have high turnover because all of those individuals to whom are then served on a caseload then have to be reassigned. And you know, obviously that’s a really tough one. And also very disruptive to client care as well.” *-Program with MOUD provider onsite*,* fewer than half of clients receiving MOUD* “Yeah, so, for example, MAT, um, because we have facility licenses was extremely regulated. They wanted to put New Jersey, wanted to be in charge of what was on the formulary and, and make some decision. They, they were not allowing us to do induction for a long time under our facility license. We had to send somebody out to somewhere else that did induction and, and like emergency room or a community-based doctor, and then they could be referred back to us for ongoing treatment, but we couldn’t start the MAT, that kinda thing. So, um, we had doctors who just said like, medically, I can’t follow the regulations by the facility license. I have, this person is suffering and it’s my medical decision that I’m gonna, uh, it start Suboxone.” *-Program with MOUD provider onsite*,* fewer than half of clients receiving MOUD* “Even though my prescriber has a controlled substance, substance thing, um, when I brought this up to her as recently as two weeks ago, she wasn’t even aware of it. Oh, okay. I don’t know how well known that change of policy is. And as a result, I don’t think there has been an uptick in prescribers because I don’t think there were honestly aware of it. And even when I made her aware of it, she, you know, she was like, well, there’s gotta be some process, some procedure. And she was afraid to prescribe anything because she, you know, any doctor doesn’t want to, you know, their, their license is their life. They, she doesn’t wanna do anything to put her license in jeopardy. And even looking online, trying to find resources for her to explain this, you can’t find them. So, you know, I think that, you know, great, they remove the waiver, but they really need to put something out to explain it. Now what does this mean? How do you prescribe it? Is it the same as every other drug? All of that needs to be explained to these prescribers so they know what to do. And then that can actually make a difference.” *- Program with MOUD partner provider offsite* “And that reflects DMHAS’s attitude that these drugs are dangerous drugs. MAT is dangerous. And that’s DMHAS’s attitude. They don’t want to let people just prescribe it. And that is awful. They, you know, there’s no reason for it not to be available. Every single doctor, you know, even now you go into a doctor’s office, they’ll ask you, how are you feeling? Are you depressed? They should be immediately, as soon as that waiver went that that happened. If they have a CDS license, they should be saying to you, how are you feeling? Are you depressed? Are you addicted to opiates? And do you wanna stop? I can prescribe Suboxone right now. If you do, that should be happening. And every doctor’s office, every time you walk in, and it’s not.” *- Program with MOUD partner provider offsite*
*Stigma and Discrimination Against MOUD*	“I just think adding that opening to other prescribers to be able to prescribe MAT is not gonna do anything cause there’s people who have it and will not prescribe it. So, that’s scary to me. But yeah, they don’t feel comfortable, and they don’t wanna deal with the constant phone calls, to be honest with you…Some prescribers are like, well, I’m not enabling this behavior until they see me, I’m not putting in a refill.” *-Program with no MOUD provider* “So I would say that it’s leadership that sets the tone. And so, we’ve been really intentional about having leadership that is fully understanding of MAT and why it’s helpful. And so, because again, there’s still some of that stigma embedded. There are counselors and in terms sometimes who come in with, um, this viewpoint that only abstinence means you’re sober. And, um, same thing. We just do a, a good amount of training about that. And sometimes you change people’s minds and, and sometimes you don’t.” *-Program with MOUD provider onsite*,* fewer than half of clients receiving MOUD* “I think a lot of, when it comes to MAT I think the biggest problem is still the stigma, um, that exists around MAT and I say unfortunately the stigma that exists in the recovery community and in also unfortunately in the treatment community is even worse than the stigma that exists in the general public. And that’s what’s, I think that’s what’s holding us back. I’ve been an advocate that, that in every one of our state contracts, it says that you cannot discriminate, you cannot turn somebody away. You cannot deny somebody MAT but our programs do it all the time. So what I would say, and I’ve said this before, is that our licensing and the division of Mental health and addiction services needs to hold people accountable, programs accountable. If they’re not providing services or if they’re turning people away or they’re denying people or they’re encouraging people to stop taking medications, they need to hold them accountable. I think it’s malpractice in today’s world.” *- Program with MOUD provider onsite*,* more than half of clients receiving MOUD* “And then in some cases they become entrenched in a philosophy of AA or NA that says you’re not clean if you’re using MAT and they want that badge of being clean without anything else…that is probably the hardest barrier to get over…” *- Program with MOUD partner provider offsite* So I think some of it just comes from that, like 12 back, that 12 step tradition of abstinence and, and people don’t see MAT as part of abstinence. And a lot of our, a lot of people in this field are people in recovery…you know, so they bring those, those kind of beliefs into the clinical practice, the abstinence beliefs.” *-Program with MOUD provider onsite*,* more than half of clients receiving MOUD* “Yeah, we still see that attitude. We still see it, um, uh, and maybe a little bit more. I mean, there’s some, we have interviewed folks who were like I don’t wanna work in the OTP program, but I’d work in your residential program cuz I don’t really believe in MAT. And it’s, I find it amazing that, you know, in the year 2000, you know, 23, that that attitude still exists, but it still exists. And a lot of the time it comes in the form of, well, yeah, I think it’s okay, but I don’t think people should be on it for a long time.” - *Program with MOUD provider onsite*,* more than half of clients receiving MOUD* “My professional networks and things, there’s still a, a huge number of clinicians who are not supportive of MOUD and treatment. And it’s mind blowing to me. I don’t understand it at all. I work with a number of agencies across the country in another capacity. And, you know, residential agencies who won’t even wanna prescribe, you know, um, psychotropic medication, much less, you know, medications for opiate disorders. Um, I mean all of them should have their licenses stripped, their agency shut down. That’s my opinion on that. I mean, I don’t understand where it comes from.” *- Program with MOUD partner provider offsite* “Some people are so rigid that, you know, they talk about, well, you’re not really clean and sober if you still, you know, if you’re taking this medication. So there are those little stigmas attached to it, but again, we are new, but those are some of the conversations that people have.” *-Program with no MOUD provider* “Like, you know, this is a medication that’s working. If it’s working, why would you wanna stop? You’re benefiting from it. And, you know, and we’ll talk about the benefits but yet there’s still that stigma that, you know, it’s, it’s a drug and I’m just replacing one drug with another drug. And so it’s a lot of education even for, for clients.” *- Program with MOUD provider onsite*,* more than half of clients receiving MOUD* “I hear that there’s some stigma in the 12 step, some of the 12 step groups around any kind of medication use, not just, you know, medications for opioid disorder, but also for some of the co-occurring issues like depression and, and so those kinds of things. So I think that there’s some shame still, um, around, you know, needing medication or this implement this, um, you know, this implied you’re weaker if you need medication to get better.” *-Program with MOUD provider onsite*,* fewer than half of clients receiving MOUD* “Sometimes it’s just outright, um, prejudices against the medication based on cultural beliefs. We see that a lot in, um, African-American communities, that they don’t want to take medications prescribed by doctors because that’s historically not been a great thing, and there’s a gut level cultural belief of this may not work out well for me, I don’t wanna do it. Um, you know, and it takes some education with them to help them get over that, right? And then in some cases, you know, they become entrenched in a philosophy of AA or NA that says you’re not clean if you’re using MAT, and they want that badge of being clean without anything else. And, you know, even if that means they never get clean, because of that, they just won’t take it even when it, you know, it’s the best thing for them and they really need to take it. And that is probably the hardest barrier to get over because that’s just a fundamental belief system that, you know, they don’t think being clean means you’re on medication and they will not budge from that opinion.” *- Program with MOUD partner provider offsite* “…We’ve had clients that have told us that the reason why they wanna stop their medication is cuz there’s family pressures from their family. There’s definitely still a lot of stigma around MAT especially… around the methadone there’s still a lot of stigma and a lot of family members [say], ‘you know, I don’t know why you’re still on that medication, you know, you should get off that medication.’” *- Program with MOUD provider onsite*,* more than half of clients receiving MOUD*
**Facilitators**	
*Education on MOUD and Harm Reduction*	“Because I think the more they understand the medication, it’s like anything else, right? The better educated a person is, the less likely they’re to be, you know, stigma, discriminatory and so forth. So, I think that education piece and really getting into why and how it works [is important].” *- Program with MOUD provider onsite*,* more than half of clients receiving MOUD* “You know, we’ve told them be honest if you’re using so that we don’t find out from your death certificate…we want to make sure that they know they can talk to us and know that it’s not going to be a punishment, they’re not going to be discharged for use of drugs…We want to help them.” -*Program with MOUD partner provider offsite* “We have, I mean, that’s built into our curriculum. It’s built into the services that we provide is, you know, um, to talk about medication…it’s in all of our programs is to talk about medications and what, how medications work. You know, we’ll get into the whole brain chemistry and help, you know, help them to understand why it works, how it works, what are the benefits.” *-Program with no MOUD provider* “Well, now in all of our vehicles we have Narcan kits in our offices, we have Narcan kits. All of our staff are trained on the use of Narcan. Our prescribers, you know, can, they can go in, we totally tell them, you know, go to a pharmacy, grab Narcan. We have family groups, that we also educate them. In the past we used to give family members Narcan. It’s really about education and kind of pushing the Narcan and even just having it available for our staff.” *-Program with MOUD provider onsite*,* fewer than half of clients receiving MOUD*
*Pharmacy Partnerships*	“… [the pharmacists] are taking some of the burden from our staff by offering to, you know, package [clients’] medications in a way that makes sense for them to hold onto them if they need them to, to deliver them when they need them to. So, it’s, it’s those kinds of strategies that I think help people to stay compliant.” *-Program with MOUD provider onsite*,* fewer than half of clients receiving MOUD* “I know something that we coordinate with our in-house pharmacy is if someone has difficulty with their medications, they can, um, instead of putting them in the bottle, they put them in like these little foil packets to help them keep track of when to take their meds.” *- Program with MOUD partner provider offsite* “We’ve kind of worked out some of those kinks with the, with the local, uh, specialty pharmacy that’s able to get it to us a little bit quicker. It used to be like a two week wait time…anytime you’re doing a daily medication, there’s a lot more risk of non-compliance than there is if you’re doing an injectable that lasts a a lot longer, which is typically why we use Vivitrol as opposed to oral Naltrexone. Just the medication compliance factor.” *- Program with MOUD provider onsite*,* more than half of clients receiving MOUD* “So our pharmacy, um, is specific to behavioral health. So, their pharmacists and techs have a level of training even around, um, how to interact with our specific population, so they’re embedded. And when they see one of our providers, you know, we’ll ask, do you want us to use our own onsite pharmacy? And the majority of our patients do use that pharmacy, and so the medications get sent right there and they can pick them up before they leave. So we have a couple of different sites. Our pharmacists or our pharmacy will deliver package and deliver to wherever the patient is. They’ll also deliver to their home if it’s a medication that they’re used to taking. So Suboxone, for example, if they’ve been on it for a little while and they’re afraid that others in the program are gonna ask them for their medications, a lot of times they’ll just have ‘em delivered right to the house. So they do a lot of tailoring to our specific population, which is great.” *-Program with MOUD provider onsite*,* fewer than half of clients receiving MOUD* “And our pharmacy helps too. They’ll give samples when they can, you know, they’ll make relationships with the pharmaceutical companies so that our clients get what they need. We’ve been pretty lucky in that way.” -*Program with MOUD provider onsite*,* fewer than half of clients receiving MOUD*
*Care Coordination*	“Even while they’re in treatment with us, we try and make sure they’re linked up with recovery coaching, especially if they’re early in recovery. We make sure that they’re solid in 12 steps if that’s something they want, we help them connect to faith-based communities if that’s what they’d like. There’s a lot of different peer initiatives around where they can go to groups and those kinds of things outside of the agency.” *-Program with MOUD provider onsite*,* fewer than half of clients receiving MOUD* “Other respondents stressed the value of advocating for their patients and individualizing care, finding that program staff can serve multiple needs of patients during their treatment. One said that they were involved in “advocating, coordinating care as much as [they] possibly can, referring clients, if [they] can’t provide it for [their patients], [they] help them find another provider.” *- Program with MOUD provider onsite*,* more than half of clients receiving MOUD* “As a matter of fact, when they go on to MAT only and they finish their clinical portions of the treatment, they’re transferred to their care manager as their primary. So [the care manager] has this good longstanding rapport with them. I do think it’s a lot of that connection, you know, that she stays engaged with them that helps them maintain [treatment].” *-Program with MOUD provider onsite*,* fewer than half of clients receiving MOUD* “If, for example, if they leave the residential programs, we make sure that they have appointments that they’re scheduled, and we follow up to make sure that they’ve made those appointments. If they haven’t, then we do a follow up. Obviously, we do lose some people. Um, yeah. We lose contact with some folks if they are referred to us…So if they go from our residential program to our outpatient program, we’re monitoring that they continue to take their medication.” - *Program with MOUD provider onsite*,* more than half of clients receiving MOUD* “And so advocating, coordinating care as much as we possibly can, referring clients if we can’t provide it for them, we’re going to help them find another provider.” - *Program with MOUD provider onsite*,* more than half of clients receiving MOUD* “So we help coordinate [recovery coaches], if that’s the need. I forgot to say that we also offer transportation to and from treatment. Along with the transportation with our CPRS, they could transport the client to the welfare office, which a lot of agencies don’t do that either. A lot of people work with Mode of Care, if you’re familiar with them, they’re just very unreliable, unfortunately. You need like two- or three-day notice to schedule an appointment. And we know most client needs are here and now. So yeah, we just do whatever we can. We don’t ever kick them out, just so you know, worst case we can give them treatment on a sliding scale or put them under a different funding that they would be eligible for. There’s almost always eligible funding. Then we would have the doctor write them, like, go to Walmart, because they have the cheapest, say they were on Suboxone, they have the cheapest plan. And then they would go there and come up with the 50 bucks or whatever it is for the prescription. So, there’s a lot of finagling. We do whatever we can.” *-Program with MOUD provider onsite*,* fewer than half of clients receiving MOUD*

## Results

### Barriers to MOUD access in specialty treatment programs

We identified three prominent themes related to barriers to provision of MOUD.

#### Theme 1: financial barriers

Financial barriers were commonly discussed by all program types. Many respondents described the high cost of MOUD, with prices varying between different formulations, medications, and insurance types. One respondent, for example, discussed the high cost of extended-release naltrexone compared to other types of MOUD:

The cost is really, really, extensive for people. They, they just can’t afford it. They can’t afford to keep up with it if they are taking it. And it’s just, its also a lot of red tape to get it because you have to find a prescriber who’s going to prescribe it and then make sure that they have it on site. (Program with no MOUD provider)

Multiple issues arose around Medicaid eligibility and coverage. Some respondents stated that many clients who lost Medicaid coverage due to Medicaid redetermining eligibility after the end of the Covid-19 Emergency were not aware they had lost coverage when they showed up to appointments. Respondents discussed restrictions around presumptive eligibility, a program that provides uninsured clients with temporary Medicaid coverage, describing that limiting the number of presumptive eligibility applications to one annually per individual enrolled in Medicaid often hindered clients’ ability to obtain MOUD and other care for clients.

Prior authorizations and insurance plans covering only specific formulations of MOUD were also discussed as an obstacle to MOUD uptake as clients were unable to get their desired formulation of MOUD. Several respondents mentioned that injectable buprenorphine (Sublocade) was a particularly challenging medication to incorporate in treatment plans as insurance did not typically cover it compared to other medications such as Vivitrol or Suboxone. In contrast, a couple respondents encountered issues having Vivitrol covered by insurance plans in comparison to other formulations. One respondent claimed,

The biggest barrier that I’ve seen is insurance coverage. If they lose coverage or, you know, they deny certain medications like some, I’ve seen some cases where Vivitrol is denied because it’s more expensive, but they will allow the oral medication than naltrexone. (Program with MOUD partner provider offsite)

#### Theme 2: shortage of buprenorphine providers

Many respondents conveyed frustration with restrictions on treatment programs and prescribers that impacted their ability to offer MOUD. Participants from programs that had no MOUD provider, an MOUD partner provider offsite, or MOUD partner provider onsite and reported fewer than half of clients receiving MOUD discussed how prescriber licensing processes and regulations delayed or deterred MOUD provision due to a shortage of available providers. One respondent discussed the extensive historical regulations and onerous training around buprenorphine prescribing which often deterred prescribers from treating individuals with OUD and resulted in a dearth of qualified prescribers of MOUD. As one respondent described:

…if I can go to my doctor and get prescribed an opiate, I should be able to go to the doctor and get prescribed MAT and it should be just as easy. There should not be any additional training, should not be any additional licensing, not be any additional credentials.there is no reason for [MOUD] to be treated the way they are. They should be like penicillin or like insulin or like any other medication. (Program with MOUD partner provider offsite)

While these interviews were conducted in the immediate period after removal of the X waiver requirement, some respondents noted a lack of awareness of changes in regulations surrounding prescriber licensing for buprenorphine. Others pointed to lack of interest or beliefs of “*enabling the behavior*” as a reason for not prescribing MOUD.

Several respondents discussed needed funds for more staff and staff retention to increase client engagement and care continuity with MOUD, and how high client turnover rates lead to a higher burden on providers.

Opinions on new opportunities to prescribe buprenorphine via telemedicine following the COVID-19 pandemic were mixed: Some were hesitant, voicing fears of diversion. Others championed the increase in access to MOUD that it provides, especially for clients unable to travel, who are sick, or otherwise unable to come in to get a prescription or a refill. They stated that post-pandemic proposed DEA rule changes requiring clients to have a physical appointment within 30 days of initiating a buprenorphine prescription would be harmful to treatment access, especially given the low risk of buprenorphine:

No one is gonna get high off Suboxone [buprenorphine]. No one is gonna get high off Vivitrol[extended-release naltrexone]. No one is gonna die from any of this stuff. Let them prescribe it. What’s the worst that happens? It’s diverted to another drug addict to help them get clean? (Program with MOUD partner provider offsite)

#### Theme 3: stigma and discrimination against MOUD

Stigma against MOUD was commonly brought up across all program types as a major barrier to MOUD uptake. Many respondents discussed the challenges of care coordination for MOUD continuation after a client leaves the treatment program given many housing programs or halfway houses don’t support MOUD. Respondents further explained how lack of appropriate clinical staff to manage MOUD in aftercare such as housing programs may contribute to the lack of access to MOUD in such settings. In addition, participants described that treatment staff who lacked medical training or had long-held abstinence attitudes often resisted the use of all types of MOUD. One respondent discussed the importance of changing these types of beliefs and the responsibility of holding programs and staff accountable:

If [the program is]not providing services or if they’re turning people away or they’re denying people or they’re encouraging people to stop taking medications, they need to hold them accountable. I think it’s malpractice in today’s world. (Program with MOUD provider onsite, more than half of clients receiving MOUD)

Methadone was commonly mentioned by most programs as being historically stigmatized, deterring many clients from initiating treatment with methadone. Respondents mentioned that internalized stigma from the clients themselves was often present, with some clients touting abstinence as the ‘true’ form of recovery and saying individuals who use MOUD are ‘weak.’ This was sometimes raised as emerging from the philosophy of 12-step or other recovery programs:

And then in some cases [clients] become entrenched in a philosophy of AA or NA that says you’re not clean if you’re using MAT and they want that badge of being clean without anything else…that is probably the hardest barrier to get over… (Program with MOUD partner provider offsite).

Finally, many respondents disclosed other sources of stigma around MOUD as coming from family members, other clients in recovery, and community members that can deter clients from initiating MOUD. One respondent discussed how families believed Suboxone was an opioid replacement which deterred the patient from using this medication. Another respondent explained,

…We’ve had clients that have told us that the reason why they wanna stop their medication is cuz there’s family pressures from their family. There’s definitely still a lot of stigma around MAT especially. (Program with MOUD provider onsite, more than half of clients receiving MOUD)

### Facilitators of MOUD access in specialty treatment programs

We identified three prominent themes related to facilitators to providing MOUD.

#### Theme 1: education on MOUD and harm reduction

Respondents from programs that had onsite providers, offsite providers, and no provider all discussed initiatives that their programs have implemented to attempt to reduce stigma toward MOUD and increase knowledge on their benefits. A program with an onsite provide with more than half of clients receiving MOUD discussed an uptick in requests for Vivitrol after providing education sessions, stating how “provid[ing] data and education around it [will] get clients that are receptive to trying those treatments.” Yet another respondent discussed how explaining the mechanism by which MOUD addresses addiction can increase awareness and acceptance of MOUD while reducing stigma surrounding its use.

Because I think the more they understand the medication, it’s like anything else, right? The better educated a person is, the less likely they’re to be, you know, stigma, discriminatory and so forth. (Program with MOUD provider onsite, more than half of clients receiving MOUD)

Building trust was also described as vital, including having open dialogues between staff and clients to reduce adverse outcomes and better prepare staff to assist clients with their needs:

You know, we’ve told them be honest if you’re using so that we don’t find out from your death certificate…we want to make sure that they know they can talk to us and know that it’s not going to be a punishment, they’re not going to be discharged for use of drugs…We want to help them. (Program with MOUD partner provider offsite)

#### Theme 2: pharmacy partnerships

Another prominent facilitator discussed by respondents that eased use of MOUD was partnering with behavioral health-specific pharmacies, where staff have a higher level of training on how to assist clients with MOUD. Respondents from programs with either an offsite provider or onsite provider explained that pharmacies often delivered medications to clients rapidly, provided samples of medications for clients, or developed relationships with pharmaceutical partners to obtain medications more efficiently. Others discussed how their partnerships with specialty pharmacies helped acquire hard-to-reach extended-release formulations of MOUD such as Vivitrol and Sublocade and reduced overall wait times for these medications. As one respondent explained:

… [the pharmacists] are taking some of the burden from our staff by offering to, you know, package [clients’] medications in a way that makes sense for them to hold onto them if they need them to, to deliver them when they need them to. So, it’s, it’s those kinds of strategies that I think help people to stay compliant. (Program with MOUD provider onsite, fewer than half of clients receiving MOUD)

Other respondents acknowledged how state funding could support pharmacy partnerships by providing financial support for initiatives such as more efficient medication delivery or covering samples of different types of MOUD for their clients.

#### Theme 3: care coordination

Some respondents described developing innovative coordination initiatives to support patients better access and continue MOUD, including hiring patient advocates or care managers. Respondents from programs that had onsite providers explained that these roles can provide support for scheduling appointments and follow-up reminders for clients, monitoring patient care plans, connecting clients with different types of care (e.g. inpatient program referrals or peer recovery coaches). The goal of these types of services was often to meet the non-clinical needs of clients to improve MOUD adherence and support treatment engagement. One respondent explained,

Even while they’re in treatment with us, we try and make sure they’re linked up with recovery coaching, especially if they’re early in recovery. We make sure that they’re solid in 12 steps if that’s something they want, we help them connect to faith-based communities if that’s what they’d like. There’s a lot of different peer initiatives around where they can go to groups outside of the agency. (Program with MOUD provider onsite, fewer than half of clients receiving MOUD)

Respondents asserted that trust between care managers and their clients is especially important given the stigma many clients face, and this trust in care managers may even help reduce relapse rates or treatment discontinuation.

So [the care manager] has this good longstanding rapport with [clients on MAT]. I do think it’s a lot of that connection, you know, that she stays engaged with them that helps them maintain [treatment]. (Program with MOUD provider onsite, fewer than half of clients receiving MOUD)

## Discussion

We interviewed leaders from specialty SUD treatment programs in New Jersey to understand what factors hinder and or facilitate uptake of MOUD in these settings.

One theme that emerged is that the costs of MOUD still remain prohibitively high for some clients despite the fact that New Jersey’s Medicaid program (the largest insurer for people in specialty substance use treatment) does not require copays for medication [15]. Consistent with other research [16, 17] respondents discussed the ongoing obstacle of high copays, deductibles, and coinsurance for commercially insured clients, as well as the burden posed by prior authorizations in both private insurance plans and Medicaid plans [18, 19]. Indeed, in a national study of clients covered under Medicaid, over 50% were found to be subject to prior authorizations for MOUD [20]. 

Respondents also described the redetermination of eligibility for Medicaid patients resulting from the end of the COVID-19 public health emergency, interruptions in coverage due to periods of incarceration, and limitations on point-of-care enrollment through “presumptive eligibility,” left some without coverage at all. Solutions to these cost barriers could include rule changes that eliminate prior authorization for certain medication formulations, prohibiting private insurance from imposing cost-sharing for MOUD, and expanding stopgap grant funding for uninsured or underinsured clients seeking MOUD.

Another common theme that emerged from the interviews was a shortage of providers willing to become licensed prescribers of MOUD at specialty treatment programs. Respondents from programs who had no provider onsite, offsite providers, or onsite providers with fewer than half of clients receiving MOUD all indicated that the workforce shortage contributes to lower MOUD uptake. Historical X-waiver requirements, combined with stigma among providers to work with MOUD, continue to make it difficult to find and retain eligible prescribers [21] and may be exacerbated in non-traditional medical settings such as specialty treatment programs [22, 23]. To address these concerns, states should consider incentives to train and retain providers, increase pay, or enhance accessibility of telehealth options. States should also consider mandatory training on substance use and mental health treatment in medical schools and residency programs [24]. 

Finally, we found that stigma continues to be a deterrent to MOUD across all program types in our study, including from providers, family members, friends, peers, other service providers and internalized stigma from clients themselves. This aligns with other research showing stigma around MOUD remains a major obstacle to its wider adoption and utilization [25, 26]. Provider-based stigma against MOUD is often cited by clients as a reason for underutilization of this treatment [27, 28], and is particularly embedded in the culture of abstinence-based treatment programs [29]. 

Even when some programs support MOUD and have even successfully hired onsite or offsite MOUD providers, clients themselves may not seek MOUD due to stigmatizing attitudes and disinterest in MOUD. Stigma may also be present in halfway houses or sober living facilities, as respondents discussed that their clients faced rejection from aftercare facilities due to their use of MOUD (despite this type of discrimination being illegal in the United States). As recovery housing has been associated with reduced substance use, improved mental health/employment outcomes, and lower rates of criminal justice involvement [30, 31], it is important to hold these facilities accountable to encourage use of MOUD.

Our study also highlights how programs have developed innovative strategies to facilitate use of MOUD.

First, programs provide education about MOUD among providers, clients, and communities, which can reduce stigma. Our findings are consistent with a recent study of key stakeholders serving people with OUD, which found that the training was successful in increasing knowledge and understanding of OUD and reducing sigma [32]. 

Second, respondents endorsed using pharmacy partnerships, emphasizing that this could be helpful for programs looking to facilitate MOUD access and retention among their clients. Historically, OUD patients have encountered numerous pharmacy barriers to MOUD like refusal to fill MOUD prescriptions or pharmacists implementing caps on the number of MOUD prescriptions filled per client [33, 34]. These actions may result from fear of DEA investigation, stigma, fear of diversion, or lack of knowledge about the role of MOUD in treatment [33, 35]. Pharmacy partnerships such as those described in this study may help address some of these barriers and improve client-pharmacy-program relationships and processes.

Finally, respondents from programs that offer onsite providers in our study often mentioned care managers as integral to supporting MOUD use through supports such as scheduling and reminders for appointments, connecting clients with resources like housing employment assistance, and managing referrals to other services. Use of peer recovery specialists as care managers may be particularly effective [36, 37], as studies have shown that care coordinators with lived experience can increase treatment retention for clients with OUD [36, 38]. 

### Limitations

This study has several limitations. First, our sample was recruited from programs that had already completed an initial survey on MOUD treatment and may not be generalizable to other programs who did not choose to participate. Second, this study was conducted shortly after the removal of the X waiver and knowledge of this policy change was not widely known or implemented at the time of the interviews, which may impact the applicability of some findings. The Covid-19 pandemic also brought about changes in MOUD-related policies, which may make the findings from this analysis unique to the changing landscape around temporary telehealth flexibilities for which the future is still unclear. Third, our interview questions did not explicitly differentiate between specific formulations of MOUD, however, we recognize that different formulations present distinct operational, logistical, and financial challenges in specialty substance use treatment settings. Thus, the barriers and facilitators identified in this study may not apply to all MOUD types and formulations. Finally, the results come from participants from New Jersey and barriers and facilitators may not be applicable to all geographic settings. Still, we believe that many of the noted barriers were not specific to state policies or circumstances and are useful for informing future policies.

## Conclusions

Understanding the barriers to uptake of MOUD in specialty treatment settings is critical for improving evidence-based MOUD access within these important touchpoints for the treatment of OUD. We identified financial issues, staffing barriers and stigma against medication use by a wide range of stakeholders continued to impede access to MOUD. We also identified strategies programs are using to overcome these barriers, including education about MOUD, forming partnerships with pharmacies, and providing support for care coordination. These findings can inform both New Jersey and other states seeking to develop policies to increase uptake of MOUD in state-licensed SUD treatment programs.

## Data Availability

All data generated or analyzed during this study are included in this published article (see Table 2).
